# Classification of Amazonian fast-growing tree species and wood chemical determination by FTIR and multivariate analysis (PLS-DA, PLS)

**DOI:** 10.1038/s41598-023-35107-6

**Published:** 2023-05-15

**Authors:** Rosario Javier-Astete, Jessenia Melo, Jorge Jimenez-Davalos, Gastón Zolla

**Affiliations:** 1 Laboratorio de Fisiologia Molecular de Plantas del PIPS de Cereales y Granos Nativos, Facultad de Agronomia, Universidad Nacional Agraria La Molina, Lima, Peru; 2 Laboratorio de Evaluación Nutricional de Alimentos, Universidad Nacional Agraria La Molina, Lima, Peru; 3 Grupo de Investigacion en Mutaciones & Biotecnologia Vegetal, Facultad de Agronomia, Universidad Nacional Agraria La Molina, Lima, Peru

**Keywords:** Carbohydrates, Infrared spectroscopy, Forestry

## Abstract

Fast-growing trees like Capirona, Bolaina, and Pashaco have the potential to reduce forest degradation because of their ecological features, the economic importance in the Amazon Forest, and an industry based on wood-polymer composites. Therefore, a practical method to discriminate specie (to avoid illegal logging) and determine chemical composition (tree breeding programs) is needed. This study aimed to validate a model for the classification of wood species and a universal model for the rapid determination of cellulose, hemicellulose, and lignin using FTIR spectroscopy coupled with chemometrics. Our results showed that PLS-DA models for the classification of wood species (0.84 ≤ R^2^ ≤ 0.91, 0.12 ≤ RMSEP ≤ 0.20, accuracy, specificity, and sensibility between 95.2 and 100%) were satisfied with the full spectra and the differentiation among these species based on IR peaks related to cellulose, lignin, and hemicellulose. Besides, the full spectra helped build a three-species universal PLS model to quantify the principal wood chemical components. Lignin (RPD = 2.27, ${R}_{c}^{2}$ = 0.84) and hemicellulose (RPD = 2.46, ${R}_{c}^{2}$ = 0.83) models showed a good prediction, while cellulose model (RPD = 3.43, ${R}_{c}^{2}$ = 0.91) classified as efficient. This study showed that FTIR-ATR, together with chemometrics, is a reliable method to discriminate wood species and to determine the wood chemical composition in juvenile trees of Pashaco, Capirona, and Bolaina.

## Introduction

The Amazon region has an area of 6.7 million km^2^ and is the largest tropical forest on the planet. Unfortunately, this region will experience the most significant forest degradation by 2030 due to indiscriminate use for activities such as scale agriculture, mining, road construction, and, to a large extent, illegal logging^1^. In 2017, the Peruvian government estimated that 37% of the wood sold in the Peruvian Amazon is of illegal origin, generating a loss of 112 million dollars annually. According to Interpol, the global illegal logging industry is worth somewhat US$152 billion a year. This situation is caused by legal non-compliance or taking advantage of loopholes in the law, affecting the forestry sector’s value chain^2^.

On the other hand, fast-growing trees such as *Calycophyllum spruceanum* (Benth.) K. Schum. (Capirona), *Guazuma crinita* Lam. (Bolaina) and *Schizolobium amazonicum* Huber ex Ducke (Pashaco) have the potential to reduce forest degradation, allowing regeneration, fertility conservation, and forest plant breeding^3–7^ to establish an industry based on wood-polymer composites in an emerging forest-based bioeconomy^7–9^. *C. sprucenum* is well known for its high primary productivity, medicinal properties, its ability to grow naturally on flooding soils, and provide storage carbon service because of its growth speed and high wood density^7^. *G. crinita* is a pioneer specie that colonizes forest gaps, has regrowth capacity, and has a short harvest cycle since poles can be harvested in 2 years and produce 160 m^3^/ha of wood volume in the sixth year^10,11^. *S. amazonicum* is a legume with the most cultivated area in the Amazon (90,000 Ha) used for soil recovery because of its tolerance to low fertility and high acidity soils^12,13^. Besides their regeneration potential and ecological services, these trees have multiple end uses and increasing demand market^11,13,14^.

In this context, the wood chemical composition is a trait that can be used in early selection since it is directly related to the wood quality and the end-use of wood. Standard wet chemical methods for wood chemical determination have been used for over a century and have proved accurate^15^. Despite the evolution of these methods over the years, they still require sample pretreatment and chemical reagents; they are also time-consuming and labor-intensive^15,16^. Therefore, alternative methods are required to speed up the process at a low cost. Fourier transform infrared spectroscopy (FTIR) is an analytical tool that addresses these problems because of its analysis speed, minimal or non-sample preparation, versatility, accuracy, non-destructiveness, and economic cost, and it requires small amounts of the sample^17–19^. Furthermore, FTIR enables the composition and structural characterization of molecules by providing information-rich spectra^18^, which allows the prediction of organic compounds (proteins, lipids, carbohydrates, and extractives^20–23^ and classifies wood species^24–26^. Through spectroscopy, breeders can select plus trees with high wood quality at a low cost^27^. However, the information contained in spectra is large and complex to interpret. Chemometrics can extract useful information from spectral data and predict chemical properties or discriminate between sample groups or species by multivariate models^15^.

Thus, FTIR coupled with chemometrics is a valuable method for classifying trees and predicting the wood chemical composition. Through pattern recognition models like PLS-DA, it has been possible to identify timber wood species and wood procedures^17,28,29^. So, it can be used to address illegal wood traffic problems and to guarantee legal provenance of timber. For phenotyping purposes, a supervised method like PLS can predict the chemical composition and physical properties of wood to increase the productivity and adaptability of the species^20,30–32^. Most of the research to predict chemical composition was almost based on single wood species. However, no Fourier transform infrared (FTIR) studies exist to build an universal model using several trees from the Amazonian region. Therefore, this work aimed to perform species classification by PLS-DA and to build three-species universal PLS models for the chemical phenotyping in juvenile trees of Bolaina, Capirona, and Pashaco.

## Results

### Wood chemical composition

Table 1 shows Capirona, Bolaina, and Pashaco wood chemical composition. Among the three species, the cellulose percentage ranged from 16.5 to 51.8%, the hemicellulose percentage from 5.5 to 35.3%, and the lignin percentage from 5.1 to 15.6%. The second most abundant compound was hemicellulose in three species and the percentage of cellulose was higher in Pashaco (44%) than Capirona and Bolaina. Table 1 also summarizes the data variation by standard deviation. Cellulose content showed a high standard deviation in all species, particularly in Bolaina (SD = 11.2). Furthermore, hemicellulose and lignin content in Bolaina had more variability than Pashaco and Capirona.

**Table 1:** Chemical wood composition in young trees.

Species	No of samples	Cellulose			Hemicellulose			Lignin		
		Average (%)	Min–Max	SD	Average (%)	Min–Max	SD	Average (%)	Min–Max	SD
Capirona	50	38.9	26.2–41.7	4.1	9.9	5.5–13.6	1.2	8.9	7.1–11.3	1
Bolaina	50	31.4	16.5–48.7	11.2	16.8	11.6–35.3	5.0	11.7	7.3–15.6	1.7
Pashaco	11	44.5	26.2–51.8	6.6	14.8	12.8–16.8	1.2	9.2	5.1–11.3	1.6

*SD* standard deviation, *Min–Max* minimum and maximum value.

### FTIR spectra

**Figure 1:**
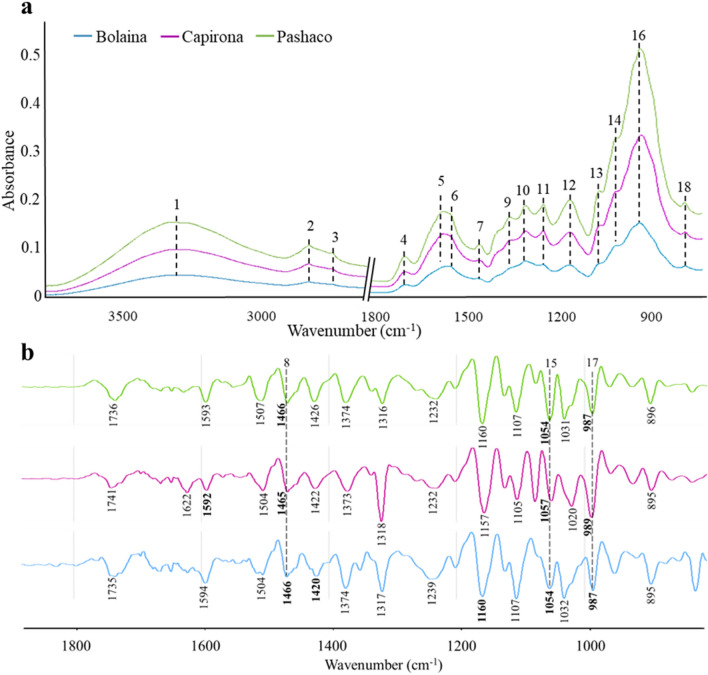
Average of raw FTIR spectra (**a**) and the second derivative of FTIR spectra (**b**) of young wood samples.

Figure 1a shows the average raw FTIR spectra obtained from all three species. These FTIR spectra evinced the presence of principal wood components with some variations in their content among the three species. Although all three species presented a similar spectral pattern, they had different absorbance intensities, and some peaks needed to be included, overlapped, or poorly defined (shoulders). Therefore, the second derivative of FTIR spectra was applied to improve peak resolution, facilitate the identification of overlapped peaks, and amplifies slight differences in spectra^26,33^. Figure 1b shows the second derivative in the fingerprint region; the bold numbers indicate peaks not seen in the raw spectra.

Peak assignment and position (wavenumber) from Fig. 1a,b are in Table 2. In Fig. 1a, the first three peaks correspond to O–H and C–H vibrations, which are present in lignin, cellulose, and hemicellulose^33^. Peak 4 corresponds to the vibration of C=O and carbonyl groups of hemicellulose^24^. Peaks 6, 7, and 9 (observed at 1605/1598, 1504/15,016, and 1418/1422 cm^−1^, respectively) are aromatic ring vibrations in lignin^24,34^. Peaks 10, 12, 13, and 14 confirmed the presence of functional groups associated with cellulose and hemicellulose^26,34^. Peaks 11, 16, and 18 are related to molecular bonds of cellulose^24,26,34^. In Fig. 1b, the second derivative in the fingerprint region evince peaks not seen in the raw spectra (bold numbers). This figure shows three typical peaks among Pashaco (1466, 1054, and 987 cm^−1^), Capirona (1465, 1057, and 989 cm^−1^), and Bolaina (1466, 1054, and 987 cm^−1^). The first peak at 1466 cm^−1^ (peak 8) is CH_2_ deformation stretching in lignin and hemicellulose^24^, and it is observed as a shoulder between peaks 7 and 9 in the raw spectra (Fig. 1a). The second peak at 1054 cm^−1^ (peak 15) is related to CO stretching in cellulose and hemicellulose^24^, while the third one is assigned to CO stretching^26^ in cellulose at 987 cm^−1^ (peak 17). In Capirona, the second derivative evinced the presence of a peak at 1592 cm^−1^ (assigned to lignin), which did not appear in the raw average spectra because of the overlapping with peak 5 (1622 cm^−1^), attributed to flavones and calcium oxalate^24^. In Bolaina, the second derivative (Fig. 1b) confirmed the absence of a 1620 cm^−1^ peak, which showed no flavones and calcium oxalate content. In the raw spectra, peaks at 1420 cm^−1^ (assigned to lignin) and 1160 cm^−1^ (assigned to cellulose and hemicellulose) are not well defined; therefore, wavenumbers were not indicated in Fig. 1a, but they were assigned correctly in the second derivative spectra (Fig. 1b).

**Table 2:** Peaks of wood samples and their assignments in FTIR spectra.

Peak number	Peak assignments	Compound	Wavenumber (cm^−1^)		
			Capirona	Bolaina	Pashaco
1	O–H vibration	All wood components	3286	3287	3337
2	C–H symmetric stretching	All wood components	2923	2922	2918
3	C–H asymmetric stretching	All wood components	2856	2854	2851
4	C=O stretching in ketone and carbonil groups	Hemicellulose	1730	1728	1732
5	C=O stretching	Flavones and calcium oxalate	1621	–	1622
6	C=C stretching of the aromatic ring	Lignin	1592	1605	1598
7	C=C stretching of the aromatic ring	Lignin	1504	1516	1504
8	CH_2_ streching	Lignin and hemicellulose	1465	1466	1466
9	Aromatic ring vibration and C–H asymmetric deformation	Lignin and polysaccharides	1418	1420	1422
10	C–H bending	Cellulose and hemicellulose	1371	1372	1371
11	CH_2_ wagging	Cellulose	1318	1319	1318
12	C–H and O–H vibration	Polysaccharides	1239	1241	1239
13	C–O–C asymmetric stretching	Cellulose and hemicellulose	1150	1160	1154
14	C–O–C stretching	Cellulose and hemicellulose	1098	1098	1102
15	C–O streching	Cellulose and hemicellulose	1057	1054	1054
16	C–O stretching	Cellulose	1021	1031	1031
17	C–O streching	Cellulose	989	987	987
18	C–H deformation	Cellulose	897	895	897

### PLS-DA models

PLS-DA is a supervised algorithm that achieves dimensional reduction with full awareness of the class labels (Y variables) used for discriminating variable selection and predictive modeling^35,36^ to classify wood species. The classification models used the PLS1-DA algorithm, which models one class at a time^35^. In a PLS1-DA regression, the Y response consists of a single variable assigned to a value of 1.0 or 0.0, denoting in-class and out-class, respectively. External validation was performed using the full spectra with MSC (multiplicative scattering correction) − 2° derivative as pretreatment, and four latent variables, previously determined by full cross-validation (Supplementary Table S1). The model performance was evaluated by R^2^, RMSEP, sensitivity, specificity, and accuracy^37,38^.

PLS1-DA has successfully achieved the correlation between wood species and FTIR spectral data, see Table 3. A good model performance presents an R^2^ close to 1 and RMSEP close to zero^30^. PLS-DA models showed good prediction capability based on these parameters because of the high ${R}_{p}^{2}$ values (0.92–0.95) and low RMSEP (0.14–0.18). Similar prediction capabilities have been reported in discriminating core-transition-outer wood of *Pinus nigra*^29^, walnut wood species^28^, and infected and normal *Aquilaria microcarpa*^39^ with values of 0.87–0.99 for R^2^ and 0.049–0.12 for RMSEP.

**Table 3:** Parameters of PLS-DA models.

Species	Latent variables	Calibration set		Test set				
		RMSEC	${\mathrm{R}}_{\mathrm{c}}^{2}$	RMSEP	${\mathrm{R}}_{\mathrm{p}}^{2}$	Sn	Sp	Acc
Pashaco	4	0.11	0.92	0.14	0.843	100.0%	100.0%	100.0%
Bolaina	4	0.11	0.95	0.15	0.91	95.8%	100.0%	98.2%
Capirona	4	0.13	0.93	0.18	0.874	100.0%	95.2%	97.3%

*Sn* sensibility, *Sp* specificity, *Acc* accuracy.

**Figure 2:**
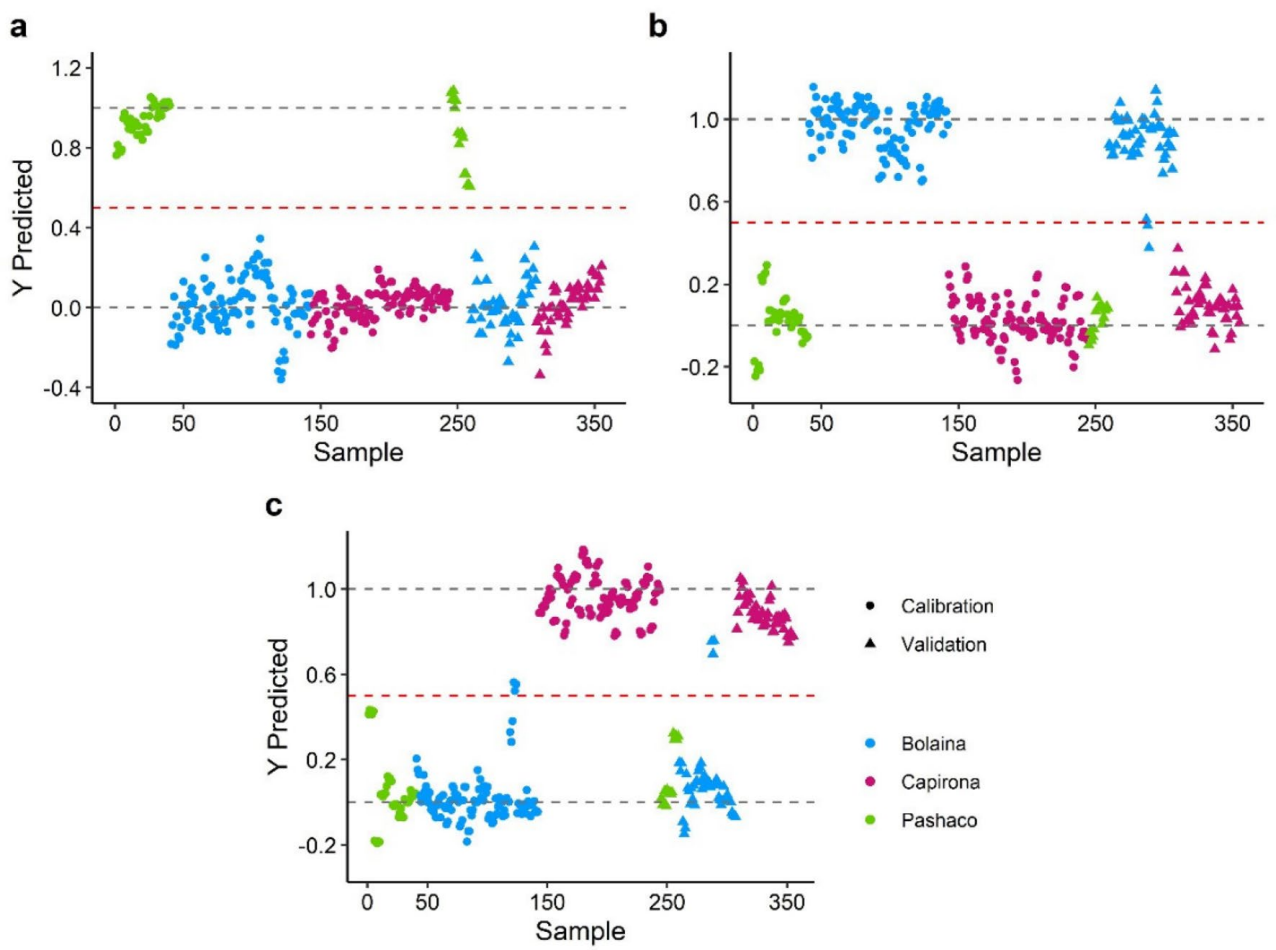
PLS-DA models.

The model performances were confirmed by accuracy, sensitivity, and specificity^40^, and these values were calculated from the discriminant plot (Fig. 2). The discriminant threshold was 0.50 (red line), depicting the boundary between Y predicted values for in-class (1.0) and out-class (0.0) samples. In Pashaco model, all samples are correctly predicted (Fig. 2a); therefore, a complete identification of classes on the validation set was reached in terms of specificity and sensitivity (100% for both). For Bolaina, a sensibility of 95.8% was achieved because of 2 false negatives (Fig. 2b), and a specificity of 100% was achieved due to no false positives. In contrast, Capirona samples were predicted correctly (100% of sensibility), but there were three misclassified samples (false positive), so the specificity obtained was 95.2%. According to Alaoui Mansori et al.^37^ and Grasel and Ferrão^41^, values of 100% for sensibility and specificity have been reported because PLS1-DA models coupled with FTIR make a good separation between classes.

X loading weights of the first and the second factor were analyzed (Supplementary Fig. S1) to identify essential IR peaks to separate wood species. Peaks at 994 and 1319 cm^−1^ are attributed to C–O stretching and CH_2_ wagging in cellulose^26,34^, while the peak at 1033 cm^−1^ is related to C–O stretching^24^ in holocellulose and lignin. Peaks at 1164 cm^−1^ and 1077 cm^−1^ are associated with C–O–C stretching of pyranose^25^ and C–O deformation in secondary alcohols and aliphatic ethers^34^, respectively. The differentiation among Capirona, Bolaina, and Pashaco is based on IR peaks related to cellulose, lignin, and hemicellulose.

### PLS models

Universal models have been reported before in other species, so a universal model to predict principal wood components for fast-growing trees is required to speed up the selection of trees in breeding programs. The FTIR spectral data and chemical composition were correlated using a universal PLS model for Capirona, Bolaina, and Pashaco. Samples were divided into modeling (2/3 of samples) and validation sets (1/3 of samples) by the block-wise selection method. The models were built using the fingerprint (1800–850 cm^−1^) and the full spectra (3700–850 cm^−1^). Before PLS analysis, spectral data were preprocessed, and the best pretreatment for each model was selected (Supplementary Table S2). Table 4 summarizes cellulose, hemicellulose, and lignin models with multiplicative scattering correction, first and second derivative as pretreatment, respectively. The predictive capability of models was evaluated based on the following statical parameters: RMSEC, RMSEP, RPD, ${R}_{c}^{2}$, and ${R}_{p}^{2}$.

**Table 4:** Universal PLS models for Capirona, Bolaina and Pashaco.

Compound	Cellulose		Hemicellulose		Lignin	
Region	FP	Full	FP	Full	FP	Full
LV	4	6	5	6	6	6
${R}_{c}^{2}$	0.89	0.91	0.78	0.83	0.78	0.84
RMSEC	3.06	2.75	2.14	1.86	0.93	0.78
${R}_{p}^{2}$	0.92	0.91	0.78	0.83	0.72	0.80
RMSEP	2.52	2.73	2.15	1.89	1.02	0.816
RPD	3.13	3.43	2.16	2.46	1.91	2.27
RMSEC/RMSEP	1.21	1.007	0.995	0.98	0.911	0.955
${R}_{c}^{2}$/${R}_{p}^{2}$	0.93	0.998	0.999	1.004	1.08	1.05

*FP* fingerprint region (1800–850 cm^−1^), *FULL* full spectra region (3700–850 cm^−1^), *LV* latent variable, ${R}_{c}^{2}$ and ${R}_{p}^{2}$ coefficient of determination of calibration and prediction, respectively, *RMSEC and RMSEP* root mean squared error of calibration and prediction, respectively, *RPD* ratio of performance to deviation.

This study reported an universal PLS model with three species to predict the main wood components. Cellulose, lignin, and hemicellulose models using the full spectra showed higher accuracy (low RMSEP) and better data fitting (R^2^) than the fingerprint region (Table 4). For the entire spectra region, the RPD value increased from 1.91 to 2.27 for lignin and from 2.16 to 2.46 for hemicellulose. In addition, both models showed slight differences between the calibration and validation set (ratio of ${R}_{c}^{2}$/${R}_{p}^{2}$ and RMSEC/RMSEP near 1), which indicates a good fit. For cellulose, the entire spectra region did not reduce the prediction error but increased the predictive power (RPD) from 3.13 to 3.43 and improved the data fitting (values of ${R}_{c}^{2}$/${R}_{p}^{2}$ = 0.99 and RMSEC/RMSEP = 1.007 are closer to 1).

**Figure 3:**
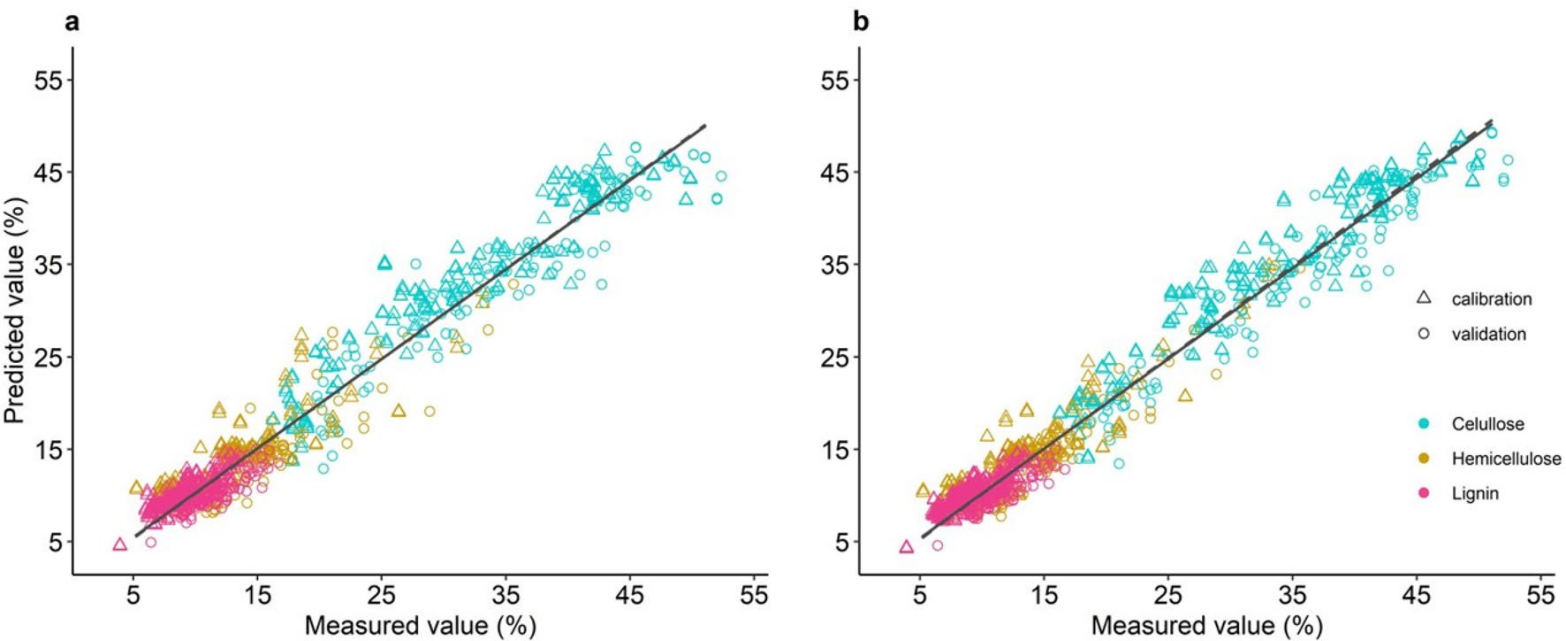
Wood principal compounds measured by Van Soest and Robertson method versus predicted by (**a**) the fingerprint region and (**b**) the full spectra region.

The full spectra region generally achieved the best performance for cellulose, lignin, and hemicellulose models (Fig. 3). This trend was also reported by Acquah et al.^22^ in samples of forest biomass. Although the fingerprint region (1800–850 cm^−1^) contains the most molecular information to build chemometrics models, the 3700 a 2700 cm^−1^ region (apparently irrelevant) improved the model efficiency. In contrast to the lignin model reported by Zhou et al.^42^, excluding wavenumbers unrelated to lignin increased the predictive power.

After establishing the full spectra as the optimum region for PLS models, the performance of lignin, cellulose, and hemicellulose models was analyzed. FTIR coupled with PLS predicts lignin content with higher accuracy (RMSEP = 0.81) than cellulose (RMSEP = 2.73) and hemicellulose (RMSEP = 1.89) content. This pattern was also reported by Funda et al.^20^, Zhou et al.^43^, and Acquah et al.^22^. Functional groups and molecular bonds are similar between cellulose and hemicellulose, while the lignin chemical structure is distinctive^22^. Therefore, the unique molecular structure of lignin increases the accuracy of the lignin model.

On the other hand, the prediction accuracy was slightly higher for cellulose, hemicellulose, and lignin models based on three species compared to models built with one single species^30^. The wood chemical composition variability may improve the predictive power (RPD) and data fitting (R^2^) in models built with more than one specie.

Due to slightly high values of RMSEP, the data correlation (R^2^) and the prediction power (RPD) of models were also included to evaluate the model performance^20,44^. According to Karlinasari et al.^44^, an RPD from 2.0 to 2.5 indicates a good prediction, and an RPD higher than 3 indicates an efficient prediction. An R^2^ from 0.81 to 0.90 indicates good prediction, and an R^2^ higher than 0.91 indicates excellent models^45^. The ${R}_{c}^{2}$ value for cellulose was higher than 0.91 with an RPD of 3.43; both values classify this model as an excellent model with efficient prediction. Hemicellulose and lignin models presented a coefficient of determination ${R}_{c}^{2}$ between 0.82 and 0.90 and RPD values of 2.27 and 2.46, respectively, so they were classified as models with good predictions. Moreover, our models showed higher predictive power (2.2 < RPD < 3.4) and better data fitting (0.80 ≤ R^2^ ≥ 0.92) than models based on one species, as reported by Acquah et al.^22^ for loblolly pine (0.80 < RPD > 2.06 and 0.74 ≤ R^2^ ≥ 0.86) and Karlinasari et al.^44^ for *Acacia mangium* Willd (1.7 < RPD > 2.3 and 0.41 < R^2^ > 0.81). This pattern was partially observed in cellulose and hemicellulose for Pinus lumber (0.93 < RPD < 2.2 and 0.90 ≤ R^2^ ≥ 0.96) by Jian et al.^46^ but not for lignin (RPD = 5.53 and R^2^ = 0.90). On the other hand, our three-species universal PLS models showed values of R^2^ (0.83–0.91) similar to models based on one specie, as reported by Funda et al.^20^ and Acquah et al.^22^. Finally, our universal PLS models proved to be efficient, like He and Hu^45^ for 116 species of wood trees, Chen et al.^25^ for hard and soft woods, and Zhou et al.^43^ for hardwood of aspen, eucalyptus, cottonwood, and poplar.

## Conclusions

In this study, the FTIR spectra of Capirona, Bolaina, and Pashaco wood at early stages were merged with chemometrics to discriminate between species and predict the cellulose, hemicellulose, and lignin content. The full spectra coupled to PLS-DA proved helpful in the discrimination between these species (0.91 ≤ R^2^ ≤ 0.94 and 0.14 ≤ RMSEP ≤ 0.18). Furthermore in the PLS-DA model, the accuracy, specificity, and sensibility was from 95.2 to 100%. The differentiation among species is related to IR peaks associated with cellulose, lignin, and hemicellulose. On the other hand, the full spectra allowed us to build a three-species universal PLS model to accurately quantify the main wood chemical components. Thus, the lignin model achieved high accuracy (low RMSEP) and was considered an excellent prediction model (RPD = 2.46, R^2^ = 0.90). The hemicellulose model is a good prediction model (${R}_{c}^{2}$ = 0.83, RPD = 2.46). Meanwhile cellulose model (${R}_{c}^{2}$ = 0.91, RPD = 3.43) was an excellent predictive model. Finally, our work could be beneficial for a quick specie determination in areas of high illegal wood traffic and the selection of plus trees based on chemical phenotyping in tree breeding programs.

## Materials and methods

### Plant material

Three fast-growing trees were used in this study, and they grew in Universidad Nacional Agraria La Molina (12° 05ʹ S, 76° 57ʹ W, and 243.7 masl). A total of 11 samples of Pashaco (1-year-old), 50 samples of Capirona (1.8-year-old), and 50 samples of Bolaina (1.8-year-old) were harvested. All samples were cut into small pieces, dried, and milled. Then samples were kept in sealed containers at air-dry moisture content until analysis.

### Wood chemical analysis

Van Soest and Robertson^47^ method determined the chemical composition of wood samples adapted to Daisy incubator and fiber analyzer AKOM 2000. Detergents separated and recovered the content of neutral fiber (lignin, cellulose, and hemicellulose) and acid fiber (lignin and cellulose). The digestion of acid fiber determined the lignin content by H_2_SO_4_. Hemicellulose content was calculated by subtracting the acid fiber from the neutral fiber and the cellulose content by subtracting lignin from the acid fiber. Finally, the chemical analysis for each sample was performed twice.

### FT-IR spectra collection

Samples were sieved (60 mesh) before spectra collection. FTIR measurements were made with attenuated total reflection (ATR) accessory in Spectrum 100 Perkin Elmer spectrometer. Spectra were recorded in the range of 4000–400 cm^−1^, with a spectral resolution of 4 cm^−1^ and 32 scans per sample. A background spectrum was collected before each sample measurement; samples were measured three times for Capirona and Bolaina and five times for Pashaco. A total of 55, 150 and 150 spectra of Pashaco, Bolaina, and Capirona were obtained, respectively. The raw spectra of samples were averaged in PEAK spectroscopy software (https://www.essentialftir.com/), and peaks were labeled with the manual peak peaking tool.

### Multivariate data analysis

#### Species discrimination

Partial least-squares discriminant analysis (PLS-DA) is a dimensionality reduction method with full awareness of the class labels (Y variables) that is used for discriminating variable selection and predictive modeling^35,36^. PLS1-DA approach (one class modeled at a time) was applied to build models with the spectral range considered as X variables and wood species (3 classes) as Y variables. Full cross-validation was performed to determine the optimal region (3700–850 cm^−1^ or 1800–850 cm^−1^), pretreatment, and the number of latent variables (Supplementary Table S1). The full spectra (3700–850 cm^−1^) were determined as the optimal region, and the spectral data were mean-centered and preprocessed with MSC (multiplicative scattering correction) combined to 2° derivative before analysis. Then an external validation was performed with 2/3 of the data (calibration set) to build models and 1/3 of the data (validation set) to evaluate them. The model performances were evaluated by R^2^ (coefficient of determination) and RMSEP (root mean square error of prediction)^48^. We also considered sensibility, specificity, and accuracy as statistical parameters. A good model shows a value of R^2^ close to 1 and RMSEP close to 0^38,48^. PLS-DA models were built on Unscramble software version 11 from Aspen technology (https://www.aspentech.com).

#### Determination of wood components

Partial least squared (PLS) is a supervised method that reduces spectral data to latent variables correlated with the response variable^49^. PLS was performed using Peak software spectroscopy (https://www.essentialftir.com/). To delete interference, the spectral data (Supplementary Table S3) were preprocessed. Before preprocessing, spectral data were mean-centered. Two regions were evaluated to build PLS models: fingerprint (1800–850 cm^−1^) and the entire infrared region (3700–850 cm^−1^). Samples were split into two sets: calibration set (2/3 data) to build models and validation set (1/3 data) to evaluate models by block-wise selection method available on Peak spectroscopy software. The calibration set contained 40, 102, and 102 spectra of Pashaco, Bolaina, and Capirona, respectively. The validation set contained 15, 48, and 48 spectra of Pashaco, Boliana, and Capirona. Jaggness statistical value, proposed by Gowen et al.^50^, was used to determine the optimal number of latent variables (Supplementary Tables S4, S5, S6). The model performance was evaluated with five statistical parameters. The root mean squared of error of the calibration set (RMSEC) and prediction set (RMSEP) measure the accuracy of a model, and it should be close to 0. RPD (ratio of performance deviation) measures the prediction power, values from 2.0 to 2.5 indicates a good prediction, and an efficient prediction has an RPD ≥ 3.0^44^. The coefficient of determination of calibration (${R}_{c}^{2}$) and prediction (${R}_{p}^{2}$) were used as a second parameter to evaluate model performance. Values from 0.81 to 0.90 indicate good prediction; values higher than 0.91 indicate excellent models^45^. To find a good-fit model, RMSEC, RMSEP, ${R}_{c}^{2}$, and ${R}_{p}^{2}$ were calculated; they should be close to 1 since a good-fit model shows a slight difference between the calibration and validation set.

### Plant material declaration

In this research, the Resolución de Dirección General 0113-2020-MINAGRI-SERFOR-DGGSPFFS granted permission to access genetic resources. This study also complies with relevant institutional, national, and international guidelines and legislation and no genotyping data has been analysed or generated during the study.

## Supplementary Information

Supplementary Figure S1.

Supplementary Table S1.

Supplementary Table S2.

Supplementary Table S3.

Supplementary Table S4.

Supplementary Table S5.

Supplementary Table S6.

## Data Availability

All relevant data are within the paper and its Supporting Information files.
