# Supplementary figures and images for: Classification of Amazonian fast-growing tree species and wood chemical determination by FTIR and multivariate analysis (PLS-DA, PLS)
Source: Sci Rep. 2023 May 15;13:7827. doi: 10.1038/s41598-023-35107-6 (PMC10185498; doi:10.1038/s41598-023-35107-6)

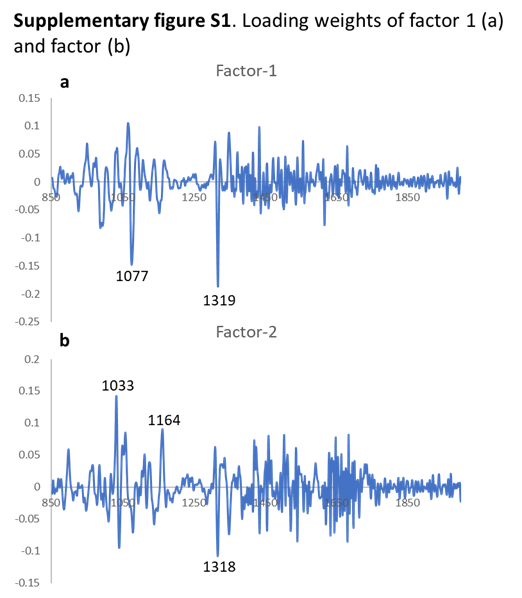

Supplement: Supplementary file 1 — Supplementary Figure S1. [file 41598_2023_35107_MOESM1_ESM.tif]
